# Time-dependent analysis of erectile dysfunction in kidney transplant recipients: insights from four distinct time periods

**DOI:** 10.1186/s12879-024-09611-7

**Published:** 2024-07-24

**Authors:** Jiashan Pan, Zhenming Zheng, Wenbo Wang, Dekai Hu, Rui Yao, Yiding Chen, Handong Ding, Jinbiao Zhong, Zongyao Hao, Guiyi Liao

**Affiliations:** 1 Department of Urology, The First Affiliated Hospital of Anhui Medical University, Jixi Road 218Th, Shushan District, Hefei, Anhui 230022 China; 2 Institute of Urology, Anhui Medical University, Hefei, China; 3 Anhui Province Key Laboratory of Urological and Andrological Diseases Research and Medical Transformation, Hefei, China

**Keywords:** Erectile Dysfunction, Kidney Transplant, SARS-CoV-2, International Index of Erectile Function Questionnaire-5

## Abstract

**Background and intention:**

Erectile dysfunction (ED) is an underappreciated clinical condition in men. This study aims to compare the dynamic changes in the distribution of ED among male kidney transplant recipients (mKTRs) in four epochs: end-stage renal disease period (ESRDp), early post-transplant period (EPTP), pre-COVID-19, and post-COVID-19.

**Methods:**

General information was gathered through interviews, follow-ups, and medical records. The International Index of Erectile Function Questionnaire-5 was used to assess erectile function. The Mann–Whitney U test and chi-square test were used to analyze differences in ED strength. Univariate and logistic regression analyses were conducted to identify risk factors for ED.

**Results:**

The database contains 230 mKTRs. In the ESRDp, 17.0% had normal erectile function, 53.5% had mild ED, 18.3% had moderate ED, and 11.3% had severe ED. In the EPTP, the distribution was 38.2% normal, 42.6% mild, 10.8% moderate, and 8.2% severe. In the pre-COVID-19 period, it was 34.3%, 47.3%, 10.4%, and 7.8%, and in the post-COVID-19 period, it was 23.0%, 45.6%, 21.3%, and 10.0%. Overall, erectile function improved after kidney transplant (KT). However, post-COVID-19, the proportion of erectile function significantly decreased compared to EPTP and pre-COVID-19 periods. Risk factors for post-pandemic ED included degree, Generalized Anxiexy Disorder-7, kidney donor type, postoperative time, hypertension and hemoglobin concentration.

**Conclusion:**

KT improves erectile function in mKTRs within 5 years, but post-SARS-CoV-2 viral infection, ED worsens due to altered risk factors. These findings inform future research for comprehensive ED prevention and management strategies in this population.

## Introduction

Over the past 70 years, kidney transplant (KT) has emerged as the preferred and cost-effective treatment for end-stage renal disease (ESRD) when compared to long-term dialysis. Moreover, significant improvements have been made in the graft and patient survival rates post-transplantation, thanks to advanced surgical techniques and the availability of innovative immunosuppressive agents [1]. As a consequence, there has been a growing demand to enhance health-related quality of life (HRQOL) as the global number of kidney transplant recipients (KTRs) continues to rise [2]. The health of KTRs encompasses the integration of physical, mental, and social well-being, with sexual function playing a crucial role in both physical and mental health. Male erectile dysfunction (ED) represents a substantial issue on a global scale, affecting a prevalence range of 11.3 to 64 percent among sexually active men [3, 4]. ED is particularly prevalent in patients with ESRD period (ESRDp), with a prevalence exceeding 80% [5]. A significant proportion of these patients also report reduced libido and a notable decline in the frequency of sexual intercourse [6]. These issues can have significant adverse effects on immune function, cardiovascular function, sleep quality, and family dynamics.

For KTRs with ED, the clinical prognosis indicates a positive trend in ED after receiving KT [7], However, current research indicates that the immune-inflammatory response driven by SARS-CoV-2 could be just a drop in the ocean when it comes to severe clinical manifestations associated with the pulmonary and cardiovascular systems. Ultimately, there is a potential emergence of clinical diseases driven by underlying multi-organ dysfunction [8]. Additionally, many sexually active individuals are facing economic and psychological pressures, as well as health concerns driven by COVID-19, inevitably experiencing impacts in various ways [9, 10]. Emerging reports within the realm of COVID-19 complications have indicated that the initial or eventual occurrence of ED could potentially serve as an alternative marker for underlying endothelial dysfunction, carrying profound significance in the prevention of cardiovascular diseases [11]. An increasing body of research suggests intricate associations between primary organic or psychogenic ED and diseases related to SARS-CoV-2 infection [12, 13].

This study aimed to gather data on ED in male KTRs (mKTRs) at different stages, including ESRDp, EPTP, pre-COVID-19, and post-COVID-19. Does KT genuinely aid in the amelioration of ED? Are recipients who experience improvements in ED prone to relapse with the prolonged duration of KT? How does COVID-19 clinically demonstrate the adverse effects on erectile function in mKTRs? We delve into the exploration of these thought-provoking academic questions.

## Materials and methods

### Data collection

**Fig. 1:**
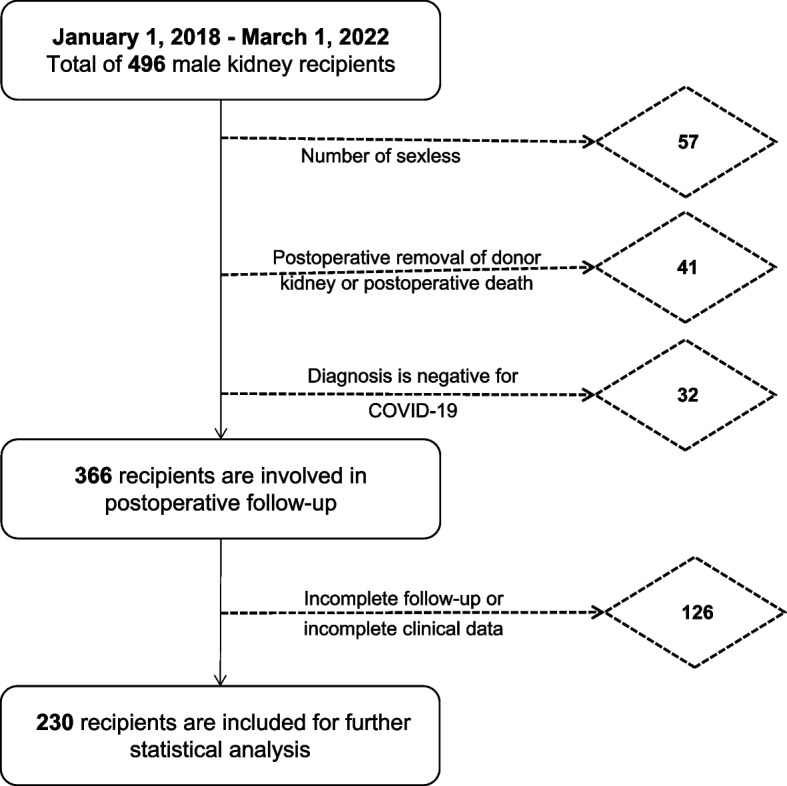
Flowchart depicting the process of data collection and analysis for ED in mKTRs. Abbreviation: ED: erectile dysfunction. mKTRs: male kidney transplant recipients

In this study, all mKTRs were collected from January 1, 2018, to March 1, 2022, and the specific screening process is shown in Fig. 1. This study was approved by our hospital ethics review (ethics number: PJ2023-10–47). The included indicators were age, postoperative time(The period from the day of completion of the kidney transplant to the end of the follow-up study), deceased donor (DD) or living donor (LD), smoking (never, former smoking, current smoking), degree (elementary, junior, high school, and above), BMI (kg/cm^2^), address (town or rural), Patient Health Questionnaire-9 (PHQ-9), Generalized Anxiexy Disorder-7 (GAD-7), type of dialysis (hemodialysis, peritoneal dialysis)(The weekly hemodialysis number variables were not included in this study because they were all three times a week in this ESRD population), tacrolimus plasma concentration, diabetes, hypertension, coronary artery disease, vascular disease and alcohol status (drinking more than once a week indicates a drinking history); Biochemical indexes: total cholesterol, triglycerides, high-density lipoprotein (HDL), non-high-density lipoprotein (nHDL), very low-density lipoprotein (VLDL), low-density lipoprotein (LDL), albumin, globulin, alanine aminotransferase, glutamate aminotransferase, creatinine, eGRF); Blood count: absolute red blood cell count (RBC), absolute white blood cell count (WBC), platelet count (PLT), neutrophils, percentage neutrophils, and hemoglobin (HB). Data collection methods: (1) When mKRTs come to the outpatient follow-up, they enter a special consultation room and complete the questionnaire content with a single self-report question. If you have any questions about the content of the questionnaire, there is a professional andrologist next to answer them. Questionnaires were filled out IIEF-5, PHQ-9, and GAD-7. (2) The demographics, medical history, and laboratory data of mKTRs were obtained from the hospital’s medical record system and examination system. (3) Exclusion criteria: mKTRs in the following cases will be excluded. 1) No stable sex life. 2) Patients who die or have allograft removed after KT 3) Diagnosis is negative for SARS-CoV-2. 4) Those who have not completed the follow-up visit completely or have lost clinical data.

### Definition

1. ED: The persistent inability to achieve and maintain an erection sufficient for satisfying sexual activity [14].
2. SARS-CoV-2 testing criteria: The nucleic acid amplification testing method is used to detect the presence of SARS-CoV-2 RNA in respiratory specimens (nasopharyngeal swabs, oropharyngeal swabs, sputum, tracheal aspirates), or other specimens. Fluorescent quantitative PCR is currently the most commonly used method for SARS-CoV-2 RNA detection.
3. ESRDp: From the day your doctor diagnoses ESRD until the day you have an allogeneic kidney transplant.
4. EPTP: Considering the potentially large variation in postoperative recovery durations among different recipients of kidney transplants, which could confound the erectile dysfunction outcomes of this study, a uniform postoperative follow-up period is therefore stipulated. Starting from the day of receiving the KT, assuming smooth recovery post-surgery, until the subsequent six months.
5. Pre-COVID-19: The time is counted from the last day of the EPTP until the test result is positive for SARS-CoV-2.
6. Post-COVID-19: It starts with a negative SARS-CoV-2 test and continues for a duration of 3 months, during which subsequent retests also yield negative results.
7. IIEF-5, PHQ-9, GAD-7: The diagnostic criteria for ED are in the form of IIEF-5 scoring. A maximum score of 25, 0–7 is severe, 8–11 is moderate, 12–21 is mild, and above 22 is normal. The degree of depression is evaluated according to the score on the PHQ-9 scale. A maximum score of 27, 0–4 is no depression, 5–9 is mild, 10–14 is moderate, and 15 or more is severe. Anxiety symptoms are evaluated on a GAD-7 scale. A maximum score of 21, 0–4 is no anxiety symptoms, 5–9 is mild, 10–14 is moderate, and above 15 is severe.

### Statistical analysis

Continuous variables are shown as mean (SD) for normally distributed variables or median [interquartile range (IQR)] for skewed variables, and categorical variables as numbers (%). To assess the overall trend and distribution differences of IIEF-5 scores among the 230 recipients in the database across four time periods, the Kruskal–Wallis test using rank sums is employed. Group comparisons are conducted using the Wilcoxon signed-rank test or the Mann–Whitney U test. The comparison of the prevalence of ED between groups was carried out using the Bonferroni method of the chi-square test. In the post-pandemic era, univariate analysis and binary logistic regression analysis methods were used to explore the risk factors leading to ED, and the regression coefficients, *p*-values, and confidence intervals for each independent variable were obtained. Data processing and charting use R code version 4.2.0, SPSS version 26.0, and GraphPad Prism version 8.0.1 software. *P* < 0.05 is shown to be statistically significant, and *p*’ < 0.008, adjusted in Bonferroni’s method, is statistically significant.

## Result

The dataset of 230 mKTRs included was grouped according to four periods, and each group was compared with two branches, normal and ED, as detailed in Table 1.

**Table 1:** Baseline characteristics of ED recipients

ED (mean ± SD/*N* (%))							
Characteristic	Total(230)^**a**^	Kidney transplant status		*P value*^***#***^	COVID19 status		*P value*^***#***^
		ESRD(191,83.0)	EPTP(142,61.7)		pre-COVID19(151,65.7)	post-COVID19(177,77.0)	
Age(year)	40.2 ± 9.7	40.7 ± 9.9	40.9 ± 9.8	*0.907*	40.6 ± 9.8	40.5 ± 9.7	*0.960*
Postoperative time(month)	33.1 ± 15.3	33.4 ± 15.3	33.3 ± 16.0	*0.944*	32.8 ± 15.5	32.2 ± 15.3	*0.724*
BMI(kg/cm^2^)	22.6 ± 3.6	22.6 ± 3.7	22.4 ± 3.4	*0.636*	22.5 ± 3.4	22.7 ± 3.8	*0.625*
Total protein(g/L)	69.5 ± 6.3	71.0 ± 10.2	62.5 ± 7.1	*0.000*	69.8 ± 4.9	69.3 ± 6.7	*0.000*
Albumin(g/L)	46.7 ± 3.5	43.0 ± 6.8	38.8 ± 5.6	*0.000*	45.0 ± 4.2	46.6 ± 3.7	*0.404*
Globulin(g/L)	23.4 ± 3.8	28.2 ± 5.5	23.7 ± 3.7	*0.000*	24.7 ± 3.9	23.3 ± 4.0	*0.000*
Alanine aminotransferase(u/L)	20.2 ± 18.0	19.4 ± 11.5	30.1 ± 31.4	*0.000*	26.4 ± 43.2	19.6 ± 16.0	*0.002*
Glutamate aminotransferase(u/L)	16.5 ± 10.0	18.4 ± 7.7	21.3 ± 16.6	*0.056*	23.9 ± 42.0	16.4 ± 9.1	*0.090*
Creatinine(umol/L)	153.7 ± 57.6	1062.2 ± 316.0	145.6 ± 46.6	*1.000*	201.8 ± 185.1	154.5 ± 59.0	*0.069*
RBC(*10^^12^/L)	4.4 ± 0.8	3.5 ± 0.9	4.2 ± 11.3	*0.000*	4.3 ± 0.9	4.4 ± 0.8	*0.005*
WBC(*10^^9^/L)	8.5 ± 11.8	6.9 ± 2.3	8.8 ± 9.2	*0.468*	7.7 ± 8.9	8.4 ± 10.5	*0.160*
PLT(*10^^9^/L)	186 ± 65.7	179.5 ± 61.0	197.7 ± 71.2	*0.000*	183.1 ± 63.9	183.6 ± 66.4	*0.534*
HB(g/L)	129.9 ± 27.4	106.7 ± 24.8	96.9 ± 23.3	*0.000*	126.5 ± 25.2	128.5 ± 29.2	*0.939*
Neutrophils(*10^^9^/L)	5.1 ± 5.5	4.8 ± 2.4	9.4 ± 41.4	*0.000*	4.4 ± 1.7	5.3 ± 6.2	*0.516*
Percent neutrophils(%)	65.7 ± 38.9	67.0 ± 12.0	72.0 ± 11.4	*0.000*	63.1 ± 10.4	67.0 ± 43.8	*0.192*
Tacrolimus concentration(ng/ml)	6.1 ± 2.3	-	13.0 ± 12.6	-	5.9 ± 2.0	6.1 ± 2.3	*0.344*
Total cholesterol(mmol/L)	4.7 ± 1.1	-	-	-	5.0 ± 5.0	4.7 ± 1.1	*0.323*
Triglycerides(mmol/L)	1.9 ± 1.1	-	-	-	2.1 ± 1.3	1.9 ± 1.1	*0.084*
HDL-C(mmol/L)	1.3 ± 0.3	-	-	-	1.3 ± 0.4	1.3 ± 0.3	*0.484*
n-HDL(mmol/L)	3.4 ± 1.1	-	-	-	3.3 ± 1.0	3.4 ± 1.1	*0.161*
VLDL(mmol/L)	0.7 ± 0.4	-	-	-	0.8 ± 0.5	0.7 ± 0.4	*0.031*
LDL(mmol/L)	3.1 ± 1.0	-	-	-	2.6 ± 0.9	3.0 ± 1.0	*0.000*
DD or LD							
DD	116(50.4)	103 (53.9)	75 (52.8)	*0.841*	81 (53.6)	95 (53.7)	*0.996*
LD	114(49.6)	88 (46.1)	67 (47.2)		70 (46.4)	82 (46.3)	
Type of dialysis							
hemodialysis	211(91.7)	174(91.1)	-	*-*	-	-	
peritoneal dialysis	19(8.3)	17(8.9)	-		-	-	
Duration of dialysis (month)	30.8 ± 35.3	32.5 ± 35.3	-	*-*	-	-	
Diabetes							
no	167(72.6)	181(94.8)	117(82.4)	*0.000*	112(74.2)	129(72.9)	*0.792*
yes	63(27.4)	10(5.2)	25(17.6)		39(25.8)	48(27.1)	
Hypertension							
no	27(11.7)	25(13.1)	29(20.4)	*0.000*	21(13.9)	16(9.0)	*0.168*
yes	203(88.3)	166(86.9)	113(79.6)		130(86.1)	161(91.0)	
Coronary artery disease							
no	218(94.8)	185(96.9)	133(93.7)	*0.000*	150(99.3)	167(94.4)	*0.037*
yes	12(5.2)	6(3.1)	9(6.3)		1(0.7)	10(5.6)	
Vascular disease							
no	219(95.2)	187(97.9)	134(94.4)	*0.060*	149(98.7)	167(94.4)	*0.056*
yes	11(4.8)	4(2.1)	8(5.6)		2(1.3)	10(5.6)	
Alcohol status^**b**^							
no	215(93.5)	173(90.6)	133(93.7)	*0.051*	144(95.4)	165(93.2)	*0.410*
yes	15(6.5)	18(9.4)	9(6.3)		7(4.6)	12(6.8)	
Smoking status^**c**^							
never	164(71.3)	138 (72.3)	110 (77.5)	*0.287*	114 (75.5)	127 (71.8)	*0.573*
forner	51(22.2)	44 (23.0)	27 (19.0)		29 (19.2)	41 (23.2)	
current	15(6.5)	9 (4.7)	5 (3.5)		8 (5.3)	9 (5.1)	
Degree							
primary school	28(12.2)	27 (14.1)	22 (15.5)	*0.439*	22 (14.6)	26 (14.7)	*0.831*
middle school	102(44.3)	86 (45.0)	65 (45.8)		68 (45.0)	80 (45.2)	
high school	41(17.8)	35 (18.3)	30 (21.1)		31 (20.5)	31 (17.5)	
> high school	59(25.6)	43 (22.5)	25 (17.6)		30 (19.9)	40 (22.6)	
Location							
town	105(45.7)	87 (45.5)	61 (43.0)	*0.638*	66 (43.7)	78 (44.1)	*0.948*
rural	125(54.3)	104 (54.5)	81 (57.0)		85 (56.3)	99 (55.9)	
Grade-PHQ9							
normal	69(30)	44 (23.0)	79 (55.6)	*0.000*	86 (57.0)	51 (28.8)	*0.000*
mild	60(26.0)	52 (27.2)	44 (31.0)		33 (21.9)	45 (25.4)	
moderate	62(27.0)	38 (19.9)	15 (10.6)		26 (17.2)	46 (26.0)	
severe	39(17.0)	57 (29.8)	4 (2.8)		6 (4.0)	35 (19.8)	
Grade-GAD7							
normal	104(45.2)	79 (41.4)	98 (69.0)	*0.001*	111 (73.5)	71 (40.1)	*0.000*
mild	85(37.0)	64 (33.5)	31 (21.8)		32 (21.2)	73 (41.2)	
moderate	34(14.8)	22 (11.5)	11 (7.7)		5 (3.3)	23 (13.0)	
severe	7(3.0)	26 (13.6)	2 (1.4)		3 (2.0	10 (5.6)	

*Abbreviation: ED* erectile dysfunction, *EPTP* early post-transplant period, *HB* hemoglobin, *HDL* high-density lipoprotein, *HDL-C* HDL cholesterol, *VLDL* Very low-density lipoprotein, *LDL* low-density lipoprotein, *DD* deceased donor, *LD* living donor

^a^Data from 230 kidney transplant populations in the post-COVID-19 era were included in the statistics

^b^As long as the frequency of drinking alcohol is more than once a week, it is considered to have a history of drinking, otherwise there is no history of drinking

^c^If you have smoked no more than 100 cigarettes in the past, it is considered never; If you used to smoke and now don’t smoke, you are considered former; If the current smoker is considered current

^#^Mean ± SD for: *P* value was calculated by weighted linear regression model.% fOr: *P* value was calculated by weighted chi-square test

**Fig. 2:**
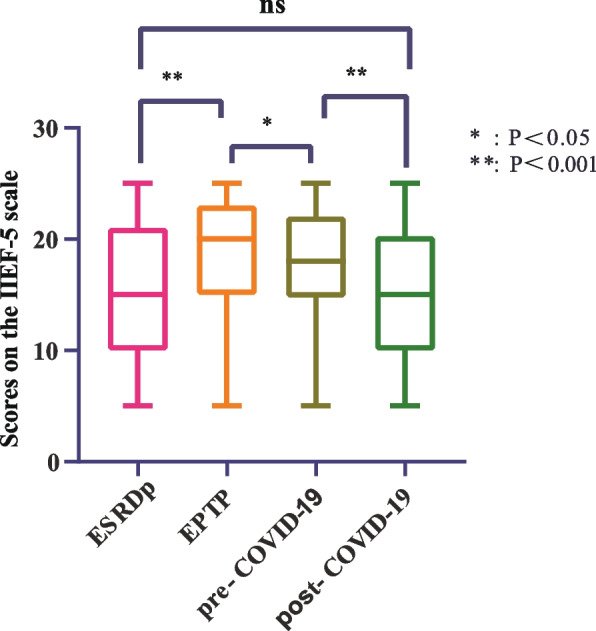
Distribution of IIEF-5 scores over four periods of mKTRs. Abbreviation: EPTP: early post-transplant period. IIEF-5: International Index of Erectile Function Questionnaire-5

**Fig. 3:**
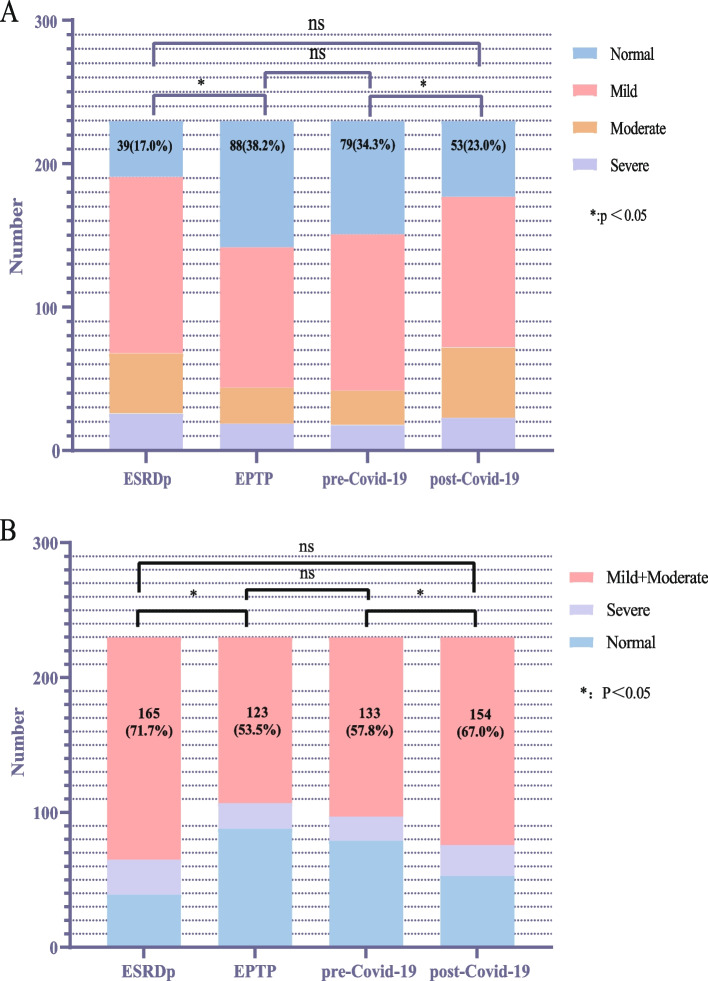
Comparison of ED severity among mKTRs in four periods. **A** Comparison of the proportion of recipients with normal erectile function across four time periods. **B** Comparison of the distribution of Mild + Moderate over four periods. Abbreviation: EPTP: early post-transplant period. legend: ‘*’ and ‘ns’ represent the statistical results of the ‘Normal’ group (Box plot A) or ‘Mild + Moderate’ group (Box plot B) over four time periods

Based on the IIEF-5 score, Table 2 shows the dynamic trend of mKTRs over four periods of ED. To compare whether there is a difference in the overall distribution of ED in the four periods, we plot a box plot for visual comparison, as shown in Fig. 2, it can be seen that there are significant statistical differences in the distribution of ED in the four periods, except for ESRDp and post-COVID-19. To compare whether erectile recovery rates improved over the four periods, we plotted stacked histograms to visually compare the mKTRs population, as shown in Fig. 3A, which showed that the normal group had a significant increase in EPTP, and by pre-COVID-19, there was no statistically different distribution between the normal group and the EPTP. This indicates that the good trend of KT-improved ED has not changed in the short term of 5 years. In the comparison of post-COVID-19 with pre-COVID-19, the proportion of normal groups is further reduced. For mKTRs for mild and moderate ED, we also plotted histogram stacked plots for four periods of the population to illustrate the statistical results, as shown in Fig. 3B. It can be seen that the proportion of the ‘mild + moderate’ group has decreased significantly in KT. This group increased significantly after suffering from the COVID-19 pandemic.

**Table 2:** ED disease profile overview

	Normal	Mild	Moderate	Severe	Total
ESRD	39	123	42	26	230
EPTP	88	98	25	19	230
pre-COVID19	79	109	24	18	230
post-COVID19	53	105	49	23	230
Total	259	435	140	86	920

*Abbreviation: ED* erectile dysfunction, *EPTP* early post-transplant period

**Fig. 4:**
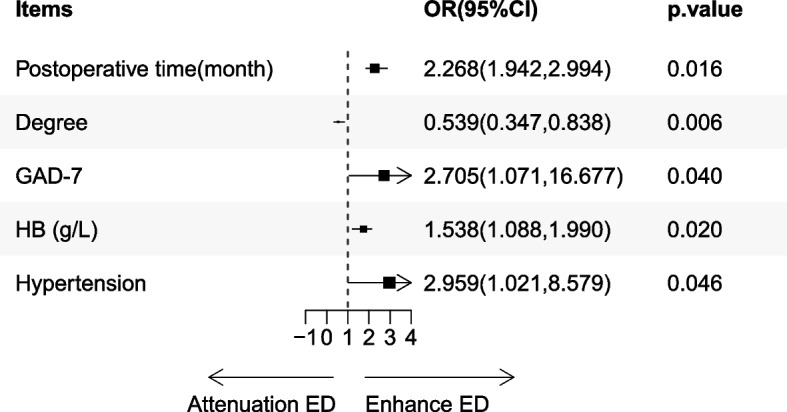
In the post-pandemic era, forest plots illustrating logistic regression results of risk factors for ED in mKTRs. Abbreviation: ED: erectile dysfunction. mKTRs: male kidney transplant recipients. HB: hemoglobin. GAD-7: Generalized Anxiexy Disorder-7

In the post-pandemic era, we performed internal analysis for risk factors that may cause mKTRs to fall into ED, and the results are shown in Table 3 and Fig. 4. The results of univariate analysis (Table 3) showed that risk factors for ED included Degree, Grade-GAD7, Grade-PHQ9, DD or LD, age (years), HDL-C (mmol/L), and hypertension. According to the Odds Ratio (OR) observations, the risk of ED in mKTRs decreases gradually with higher levels of education and HDL-C content. However, it is positively correlated with anxiety, depression, kidney donation from deceased donors, and hypertension. The multivariate logistic regression analysis in Fig. 4 showed that five factors, including degree, HB (g/L), postoperative time (month), Grade-GAD7, and hypertension, were strongly associated with the occurrence of ED.

**Table 3:** Univariate analysis of risk factors associated with ED in the post-pandemic era

Characteristics	OR (95%CI)	*P* value^#^
**Degree**	**0.678 (0.497–0.926)**	**0.014**
**Grade-GAD7**	**1.087 (1.011–1.169)**	**0.024**
**Grade-PHQ9**	**1.099 (1.091–1.110)**	**0.028**
**DD or LD**	**0.566 (0.303–0.958)**	**0.045**
Age (year)	1.012 (0.980–1.045)	0.458
Smoking status^**a**^	0.805 (0.493–1.314)	0.385
Postoperative time (month)	0.984 (0.965–1.004)	0.117
HB (g/L)	0.991 (0.979–1.004)	0.165
**HDL-C (mmol/L)**	**0.407 (0.166–0.999)**	**0.049**
Total protein (g/L)	0.964 (0.904–1.028)	0.260
PLT (*10^^9^/L)	0.998 (0.993–1.002)	0.312
Total cholesterol (mmol/L)	0.868 (0.661–1.138)	0.305
Glutamate aminotransferase (u/L)	0.992 (0.977–1.008)	0.338
Neutrophils (*10^^9^/L)	1.095 (0.905–1.326)	0.349
Address	1.318 (0.713–2.438)	0.379
Percent neutrophils (%)	1.011 (0.985–1.038)	0.402
LDL (mmol/L)	0.918 (0.678–1.242)	0.577
WBC (*10^^9^/L)	0.995 (0.972–1.019)	0.680
Albumin (g/L)	0.982 (0.899–1.072)	0.682
Triglycerides (mmol/L)	1.061 (0.798–1.410)	0.684
Creatinine (umol/L)	1.001 (0.996–1.007)	0.714
Alanine aminotransferase (u/L)	0.995 (0.966–1.025)	0.754
Globulin (g/L)	0.988 (0.912–1.070)	0.761
n-HDL (mmol/L)	0.960 (0.726–1.269)	0.773
BMI (kg/cm^2^)	1.013 (0.928–1.105)	0.777
Tacrolimus concentration (ng/ml)	1.004 (0.878–1.148)	0.954
RBC (*10^^12^/L)	1.000 (0.684–1.461)	0.999
Diabetes	0.943 (0.476–1.867)	0.865
**Hypertension**	**2.635 (1.138–6.101)**	**0.024**
Coronary artery disease	1.527 (0.324–7.196)	0.593
Vascular disease	3.114 ( 0.390–24.890)	0.284
Alcohol status^**b**^	1.212 (0.329–4.466)	0.772

*Abbreviation: ED* erectile dysfunction, HDL high-density lipoprotein, *HDL-C* HDL cholesterol, *VLDL* Very low-density lipoprotein, *LDL* low-density lipoprotein, *DD* deceased donor, *LD* living donor

^a^As long as the frequency of drinking alcohol is more than once a week, it is considered to have a history of drinking, otherwise there is no history of drinking

^b^If you have smoked no more than 100 cigarettes in the past, it is considered never; If you used to smoke and now don’t smoke, you are considered former; If the current smoker is considered current

^#^Mean ± SD for: *P* value was calculated by weighted linear regression model.% fOr: *P* value was calculated by weighted chi-square test

## Discussion

In the pathogenesis of ESRD with high-incidence ED, it is currently believed to be caused by multiple factors. Various factors contribute to its development, including abnormalities in the hypothalamic-pituitary–gonadal axis, disturbances in the autonomic nervous system, peripheral neuropathy, endothelial dysfunction, anemia, secondary hyperparathyroidism, medication effects, and psychological factors like stress and depression. These factors collectively play a role in the occurrence of ED, albeit to varying degrees [15]. The results of our study (Fig. 4) also found that HB and GAD7 played an important role in the deterioration of ED in mKTRs. Immunosuppressants and antihypertensive medications are involved in the occurrence of ED in mKTRs. Specifically, calcineurin inhibitors such as cyclosporine and tacrolimus, mTOR inhibitors, and corticosteroids may impact endothelial function and/or testicular function/structure [16]. This supports our research finding that kidney transplant therapy in ESRD patients lowers the rate of ED, though it still exceeds that of the general population.

From an epidemiological perspective, as KTRs live longer than ever, it is crucial to prioritize their HRQOL. Among them, the incidence of ED in mKTRs was generally between 54 and 66% [17, 18], and our center was 61.8%, which improved the ED status of mKTRs by 21.2% compared with 83% during the ESRDp. These findings further support previous reports which suggest that KT significantly improves ED [19]. However, the situation is not entirely optimistic. During the questionnaire collection process, it was discovered that many mKTRs with severe ED reported that their ED persisted even after undergoing KT, with little improvement observed. In the post-pandemic era, the reported trends of ED among mKTRs remain unknown. The initial findings from our center indicate a prevalence rate of 77%. When compared to the ESRDp group, there were no significant statistical differences observed in the distribution of the average International Index of IIEF-5 scores, as illustrated in Fig. 2. Additionally, there were no statistically significant differences observed in the proportion of individuals with normal erectile function when compared to the ESRDp group, as shown in Fig. 3A. Has the impact of the COVID-19 pandemic on mKTRs in terms of erectile function offset the improvement in KT? This also requires multi-center further verification.

In the treatment of ED, various approaches are available, including medication and non-pharmacological interventions. In terms of pharmacological treatment, literature reports indicate that Tadalafil therapy has demonstrated significant efficacy in individuals with ESRD and ED. It has been shown to improve both erectile and ejaculatory functions, offering a valuable reference for the treatment of ED in mKTRs [20]. In addition, numerous clinical studies have reported that testosterone replacement therapy can significantly enhance both the structural integrity and functional performance of the corpora cavernosa in patients diagnosed with ED. This therapeutic approach has been shown to promote improved erectile function, thereby addressing a key pathological component of ED [21, 22]. Furthermore, subsequent research has emphasized the necessity for stringent clinical guidelines to ensure that testosterone replacement therapy is administered exclusively to male patients who present with clear indications of ED, thereby optimizing treatment efficacy and minimizing potential risks associated with inappropriate hormone therapy [23]. Psychological therapy is another option. Extracorporeal low-intensity shockwave therapy is an external treatment modality. Tacrolimus has shown no significant effect on the ED of mKTRs before and after COVID in our center’s statistical study (*p* = 0.344), but it has been reported in the literature that Tacrolimus can significantly prolong the peak concentration of sildenafil in mKTRs and prolong the elimination half-life of sildenafil [24]. It has been proven that mild and moderate ED show better treatment outcomes with medication or other methods [25, 26], so we focused on this demographic. According to the stacked bar chart in Fig. 3B, the ‘mild + moderate’ group represents the majority of cases of ED, and its proportion varies inversely with the number of individuals in the normal group across the four time periods. In the post-pandemic era, there is no statistically significant difference in the number of individuals with ‘mild + moderate’ ED compared to the ESRDp group. This suggests that in the post-pandemic era, there is an increasing incidence of ED among mKTRs, and a significant portion of the ED population may have transitioned from the normal group. To enhance the HRQOL for mKTRs, andrologists, and kidney transplant specialists should allocate more energy and time towards addressing the treatment of mild or moderate ED.

Understanding the risk factors associated with ED is a critical theoretical basis in the dimension of preventing recurrence or worsening of ED. The risk factors influencing ED encompass both organic factors and psychogenic and relationship factors. Previous literature has reported that lifestyle factors such as smoking, alcohol consumption, obesity, and excessive intake of red meat can contribute to the occurrence of ED [27, 28]. Our study also found a strong correlation between epidemiological data and ED, such as the degree. Education is inversely correlated with ED, possibly because people with low levels of education pay less attention to health care, quality of life, and sexuality [29]. Analyzing from the perspective of psychogenic and relationship factors, The widespread fear of mKTRs and the global population towards COVID-19, along with the uncertainty of the future, financial and economic losses, and reduced social support during lockdown, have exacerbated psychological distress, depression, and anxiety among individuals in the population [30]. Both of these conditions are closely associated with the occurrence and development of ED [31]. Previous research had primarily focused on the quantitative impact of depression on ED. However, in the post-pandemic era, this focal point may shift. Given the global spread of COVID-19, sensitive populations like KTRs may be more significantly affected by anxiety. Furthermore, our center has confirmed this observation by analyzing the impact of PHQ-9 and GAD-7 on ED. As seen in Table 3 and Fig. 4, the severity of anxiety is positively correlated with the incidence of ED in mKTRs, while the correlation between depression and ED is not significant.

Naturally, there are several limitations to this study. Firstly, as this is the first study to investigate the data regarding ED in mKTRs in the post-pandemic era, the results need to be validated and supplemented by multicenter studies. Secondly, the scoring used to diagnose ED relied on a single self-report question. Due to the wide period covered by the four scenarios, there may be biases in patients’ retrospective reports of erectile function in the previous three scenarios. Finally, the impact of testosterone on ED and the effectiveness of testosterone replacement therapy for ED treatment are currently hotly debated topics in the medical field. It is important to highlight that this study did not gather data on hormonal markers in mKTRs. As a result, further investigation is necessary to explore the potential relationship between ED and these hormonal factors within specific patient groups.

## Conclusion

This study has revealed the dynamic trends in the distribution of ED among mKTRs during four crucial periods. KT can improve erectile function in mKTRs and appears to be effective within 5 years. Additionally, it highlights the worsening of erectile function in mKTRs following the impact of COVID-19. These findings provide a foundation for further research, aiming to develop comprehensive strategies for preventing and managing ED in this patient population.

## Data Availability

The data underlying this article will be shared on reasonable request to the corresponding author.
